# Medicinal Cannabis for Paediatric Developmental, Behavioural and Mental Health Disorders

**DOI:** 10.3390/ijerph20085430

**Published:** 2023-04-07

**Authors:** Daryl Efron, Kaitlyn Taylor

**Affiliations:** 1 Murdoch Children’s Research Institute, 50 Flemington Rd, Parkville, VIC 3052, Australia; kaitlyn.taylor@mcri.edu.au; 2 The Royal Children’s Hospital, 50 Flemington Rd, Parkville, VIC 3052, Australia; 3 Department of Paediatrics, The University of Melbourne, Parkville, VIC 3052, Australia

**Keywords:** medicinal cannabis, cannabidiol, paediatrics, developmental disorders, mental health

## Abstract

Parents of children with developmental, behavioural and mental health disorders are increasingly asking whether medicinal cannabis might be a therapeutic option for their child. This paper presents the current evidence for medicinal cannabis in this population. Preliminary evidence from open-label studies suggests the potential for medicinal cannabis to ameliorate some symptoms in children with autism spectrum disorder. However, only one double-blind placebo-controlled trial has been completed, with inconclusive findings. Synthetic, transdermal cannabidiol gel has demonstrated efficacy for reducing social avoidance in a sub-group of children with Fragile X syndrome. Studies of medicinal cannabis are planned or underway for children and/or adolescents with autism, intellectual disability, Tourette’s syndrome, anxiety, psychosis, anorexia nervosa and a number of specific neurodevelopmental syndromes. High quality evidence from double-blind placebo-controlled trials is needed to guide clinical practice.

## 1. Introduction

There is increasing interest in medicinal cannabis (MC) as a treatment for paediatric developmental, behavioural and mental health disorders [1]. Psychotropic medications including anti-depressants, psychostimulants and antipsychotics are often prescribed to treat behavioural and mental health problems in children with developmental disabilities [2,3,4]. Unfortunately, these medications carry a high risk of adverse effects, including sedation, changes in mood, appetite and cognition, metabolic syndrome and extrapyramidal effects [5,6]. Therefore, it is not surprising that both parents and paediatricians are interested in novel interventions that may have a superior safety profile, such as MC.

The endocannabinoid system appears to play an important role in neurodevelopment and behaviour [7], and there is some pre-clinical support for a physiological mechanism for MC in treating neurodevelopmental disorders. For example, alterations in endocannabinoid system functioning have been observed in autism spectrum disorder (ASD), Fragile X and Tourette’s syndrome [8,9,10,11]. Immune dysregulation and neuroinflammation are believed to be key cellular mechanisms underpinning ASD [12], and MC appears to have anti-oxidant, anti-inflammatory, and neuroprotective effect, so may be beneficial in patients with neurodevelopmental disorders [13]. Furthermore, problematic anxiety is a common and impairing symptom in patients with neurodevelopmental disorders, and there is good evidence from animal studies that MC has anxiolytic effects, via activation of the cannabinoid type 1 receptor [14]. Therefore, there is biological plausibility for MC as a potential treatment for childhood developmental and mental health disorders.

A number of cellular mechanisms appear to be involved in the pharmacological modulation of the endocannabinoid system affected by MC, which may influence symptomatology in neurodevelopmental and mental health disorders. The main element of the endogenous cannabinoid system in the brain comprises the cannabinoid type 1 receptor, which is present at high levels on inhibitory (GABAergic interneurons) and to a lesser extent on excitatory (glutamatergic) terminals [15], and may play an important role in the modulation of mood and behaviour. MC also appears to exert an interactional effect on related neurotransmitter pathways, including the serotonergic and dopaminergic systems [16].

Paediatricians have become aware of the proven benefits of cannabidiol (CBD) for seizure control in children with Lennox Gastaut and Dravet syndromes [17,18,19], and in a retrospective chart review, one third of parents of children with refractory epilepsies treated with CBD reported improvements in alertness and behaviour [20]. Furthermore, there are many anecdotal reports on the internet from parents of children with developmental disorders (particularly ASD) describing improvements with the administration of either unregulated or prescribed cannabis products. Reported effects have included reductions in anxiety, agitation and aggression, in some cases have resulted in the ability to reduce or cease other psychotropic medications, thereby reducing the risk of adverse effects. 

In this article, we review the evidence base for MC for paediatric developmental, behavioural and mental health disorders. The literature was selected for inclusion from systematic reviews in this field of study, keyword searches, and follow-up of trials marked as complete in trial registries. Single-case studies and conference abstracts were excluded. Searches of ongoing trials were conducted in the Australian and New Zealand Trial Registry (ANZCTR), Clinicaltrials.gov, ISRCTN and the EU Clinical Trials Register.

## 2. Autism Spectrum Disorder

ASD is a developmental disorder affecting approximately 1% of children [21]. A substantial proportion of children and adolescents with ASD have comorbid mental health disorders, including anxiety disorders and obsessive compulsive disorder [22]. Distress resulting from these problems can be expressed with challenging behaviour including irritability, aggression, and self-injury, such as head banging, arm biting, and skin scratching [23,24]. These behaviours can pose a risk to self and others, and have a major impact on the individual’s daily functioning, capacity for participation and quality of life. Up to half of all children with ASD are treated with psychotropic medications [25]. In recent years, MC has begun to be studied as an alternative treatment option.

A number of uncontrolled retrospective or prospective case series have been published, exploring MC as a treatment in ASD (see Table 1) [26,27,28,29,30,31]. Most studies have assessed both the core symptoms of ASD (e.g., social communication) and common co-morbid symptoms such as disruptive behaviour or anxiety. While not all participants appear to benefit from medicinal cannabis, and a minority report worsened symptoms or intolerable adverse events, most of these studies concluded that medicinal cannabis benefited a substantial proportion of participants. Improvements have been reported in behaviour, sleep, social function and communication. A recent systematic review of MC in ASD identified that improvements had been reported in a broad range of outcomes including anxiety, agitation, aggression, and self-injurious behaviour, as well as sleep, cognition, attention, social interaction, and language, but concluded that randomised placebo-controlled trials were needed to clarify these findings [32]. 

Adverse events are largely described as mild; most commonly, they are appetite changes, somnolence, or worsened behavioural or anxiety symptoms. Only one study reported a serious adverse event, psychosis, considered to be related to the higher THC content given to that participant. Although these preliminary reports are promising, several of these studies are limited by small sample sizes or insufficient follow-up of participants who discontinued treatment, and all are limited by being open-label or observational studies. Double-blind placebo-controlled trials are needed to test efficacy scientifically.

To date, only one blinded, placebo-controlled trial of MC to treat symptoms associated with ASD in children has been published [33]. Participants (*n* = 150) aged 5–21 years were randomised to receive one of three study medications: pure CBD and THC in a 20:1 ratio, whole plant CBD and THC in the same ratio, or placebo. The initial dose was 1 mg/kg/day CBD, and the dose was increased by 1 mg/kg/day CBD every other day, up to 10 mg/kg body weight per day CBD for children weighing 20–40 kg, or 7.5 mg/kg/day CBD for weight > 40 kg, to a maximum of 420 mg CBD and 21 mg THC per day. The average CBD treatment dose was 5.7 mg/kg/day in the whole-plant extract arm and 5.9 mg/kg/day in the pure cannabinoids arm. Although designed as a cross-over trial, only data from the first treatment period were analysed due to a treatment order effect, which limited sample size. The results were mixed across the two co-primary outcome measures. Changes in behaviour symptoms, as measured by a parent questionnaire (Home Situations Questionnaire), were not significantly different between the groups. However, disruptive behaviour (Clinical Global Impression-Improvement scale) was much or very much improved in 49% of participants in the whole-plant extract group compared to 21% in the placebo group (*p* = 0.005). A positive response was rated in 38% of participants in the pure cannabinoid group, which was not statistically significant compared to placebo (*p* = 0.08). Improvement in ASD symptoms (Social Responsiveness Scale) was significantly greater in the whole-plant extract treatment group but not the pure cannabinoid group, relative to placebo. Neither the pure nor the whole-plant cannabinoid was superior to placebo in improving parents’ stress or participants’ sleep disturbances in this sample [34]. There were no treatment-emergent serious adverse events. Common adverse events included somnolence, decreased appetite, weight loss, tiredness, euphoria and anxiety, although somnolence was the only adverse event which occurred statistically more frequently in the treatment groups than the placebo group.

In clinical practice, choice in product and dose is poorly informed by the current literature. To date, studies in ASD that have involved use of CBD predominant products have commonly had a 20:1 CBD:THC ratio, with or without minor cannabinoids. The doses used have varied substantially; one study reported a maximum daily CBD dose of only 40 mg/day [28], while others have used doses of up to 10 mg/kg/day (maximum 420 mg/day) [33]. In addition, several studies have used individualised up-titration depending on tolerability and perceived clinical benefit, further clouding interpretation of the optimal dose. With the potential risk of THC to the developing brain being unclear, some medicinal cannabis manufacturers have drawn criticism due to their marketing of high-THC products (e.g., 1:1 ratio of CBD and THC) for young patients with ASD [35]. While a company’s white paper describes clinical improvement in ASD patients who were prescribed their 1:1 CBD:THC product [36], these findings have not been published in a peer-reviewed journal, and the product’s purported benefits have not been assessed in a rigorous placebo-controlled trial. Although infrequent, there have been reports of hallucinations in children taking MC with a relatively higher THC-to-CBD ratio, including in three patients (one with an associated suicide attempt) taking Nabiximol for spasticity [37], and one in a retrospective case series in ASD [26]. Another consideration is delivery method. The majority of published papers report outcomes from naturally derived MC products administered orally; however, one company reported behaviour and anxiety improvements in patients with ASD who used a synthetic transdermal CBD gel in an open-label trial (the results of which were reported in conference presentations but not peer-reviewed) [38]. Further research is necessary to clarify the optimal therapeutic dose of CBD, the additional risks and benefits of THC and minor cannabinoids, and differences in clinical benefit between whole-plant, isolate and synthetic products.

There are ten currently registered trials of MC in children and adolescents with ASD, including three large-scale double-blind placebo-controlled trials (see Supplementary Table S1). Seven of these trials use either pure CBD (e.g., Epidiolex) or CBD-predominant (e.g., CBD and THC in a 20:1 ratio) products; however, one trial will use cannabidivarin (CBDV), which is a homolog of CBD with similar pharmacological properties [39].

## 3. Intellectual Disability

Children and adolescents with intellectual disability (ID) have high rates of comorbid mental health disorders which are often highly impairing [40]. Psychotropic medications are often prescribed, with variable effects and a high risk of adverse effects [2,3]. Furthermore, patients with ID are at particularly high risk of adverse effects [41], while being less able to report subjective experiences, rendering the use of these medications challenging in this patient group. Novel and potentially safer medications such as MC could provide therapeutic advantages if shown to be effective. 

Apart from a case series report of Dronabinol in 10 adolescents with ID and self-injurious behaviour [42], there have been no studies of MC published in samples defined by having ID. Several of the currently registered trials of ASD exclude patients with ID, and this subgroup (idiopathic ID without ASD diagnosis) is often overlooked in clinical trials, despite experiencing as significant behavioural problems as patients with ASD. An initial pilot study aimed to assess the feasibility and acceptability of a randomised placebo-controlled trial investigating CBD as treatment for severe behaviour difficulties in children with intellectual disability (IQ below 70), with or without ASD [43]. Eight children aged 8 to 16 years old were given either CBD (20 mg/kg/day) or placebo for 10 weeks. CBD was well tolerated, with no serious adverse events or study withdrawals. The study design was found to be feasible and acceptable to families. Although not able to make conclusions regarding efficacy, all three participants who received CBD and completed the outcome questionnaires reported clinically significant improvement in severe behaviour (Aberrant Behavior Checklist-Irritability scale), whereas improvement was not reported in any of the participants who received the placebo. This pilot study informed the design of a multi-site randomised placebo-controlled trial, which is currently recruiting children aged 6 to 18 with intellectual disability and severe behaviour problems, using pure CBD (10 mg/kg/day). 

## 4. Neurodevelopmental Syndromes

Parents of children with a variety of genetic syndromes with associated neurodevelopmental disability are increasingly inquiring about the use of MC. This occurs commonly after hearing reports of observed benefits from other parents via syndrome-specific social media groups.

MC research is underway in a number of specific neurodevelopmental syndromes. In children with Fragile X syndrome (FXS), an open label study of Zynerba synthetic transdermal CBD gel (250 mg) reported a clinically meaningful reduction in anxiety and behaviour symptoms [44]. This led to a phase 2/3 randomised, double-blind, placebo-controlled efficacy and safety study in 212 children aged 3 to <18 years. Participants were treated for 12 weeks with Zynerba CBD gel—250 mg/day or 500 mg/day for participants ≤35 kg or >35 kg, respectively. Statistically significant group differences were not found between participants treated with placebo or Zynerba gel on the primary outcome measure of social avoidance, as measured on the Aberrant Behavior Checklist-Community FXS specific subscale, nor on the secondary outcomes including irritability, socially unresponsive behaviour and improvement ratings from the Clinical Global Impression-Improvement and Caregiver Global Impression-Change measures. However, pre-planned analyses were conducted in patients with at least 90% methylation of the FMR1 gene, associated with the most severe phenotype. In this subgroup, statistically significant improvements in social avoidance were seen in the CBD group relative to the placebo, and similarly in the caregivers’ global impression of change ratings [45]. Treatment-emergent adverse events were all considered mild or moderate, most commonly application site pain. A phase 3 trial (Clinicaltrials.gov: NCT04977986) and an open-label extension trial (Clinicaltrials.gov: NCT03802799) are underway to confirm the results of the phase 2/3 trial using a broader dose range (750 mg/day for patients weighing >50 kg).

Registered trials in MC and other specific neurodevelopmental syndromes include:

1. An open-label study of Zynerba transdermal CBD gel in 20 children with 22q11.2 deletion syndrome (Clinicaltrials.gov: NCT05149898), with safety as the primary outcome and a range of behavioural and mental health secondary outcomes;
2. A blinded, randomised, placebo-controlled study of CBDV in 26 patients with Prader–Willi syndrome aged 5 to 30 years old, with irritable behaviour as the primary outcome (Clinicaltrials.gov: NCT03848481);
3. A placebo-controlled *n*-of-1 series investigating the effectiveness of CBD on behavioural problems in patients 6 years and older with tuberous sclerosis complex, Fragile X syndrome and Sanfilippo syndrome (Clinicaltrialsregister.eu: 2021-003250-23).

A trial of CBD in patients with Rett Syndrome was terminated due to enrolment challenges and the COVID-19 pandemic (Clinicaltrials.gov: NCT03848832), while a trial of CBD in patients with Prader–Willi Syndrome was terminated due to a “change in corporate priorities” (Clinicaltrials.gov: NCT05098509).

## 5. Attention Deficit Hyperactivity Disorder 

Attention deficit hyperactivity disorder (ADHD) is the most common neurodevelopmental disorder, with a prevalence of approximately 5% [46]. Most children with significant ADHD are treated with medication, most commonly the psychostimulants [47,48]. While these medications are often effective for symptom reduction, adverse effects are common, and the long-term benefits and risks of stimulant medication in ADHD remain uncertain [49]. There is a long history of complementary and alternate medicine use in ADHD [50], and interest in MC as a treatment option has emerged in recent years [51]. 

People with ADHD are at increased risk of recreational cannabis use and cannabis use disorder compared to the general population [52]. In spite of this, there is a perception that cannabis may be therapeutic for ADHD symptoms [53], with some suggesting that young people with ADHD may be self-medicating with cannabis, both for core symptoms of ADHD (e.g., hyperactivity, impulsivity) as well as improvement in medication side effects (e.g., anxiety) [54]. A randomised controlled trial in adults with ADHD provided preliminary support for the role of Sativex (1:1 CBD:THC) in improving hyperactivity/impulsivity and a cognitive measure of inhibition; however, the sample size was small (*n* = 30) and the results were no longer significant after adjusting for multiple testing [55]. 

An ongoing study aims to monitor perceived effectiveness and pharmacokinetics in a sample of 10–20 patients aged 12 to 25 who are currently prescribed MC for treatment of ADHD with features of oppositional defiant disorder [56]. There are no published or registered randomised placebo-controlled trials in paediatric patients with ADHD. Despite the lack of evidence, MC is increasingly prescribed in Australia for children for ADHD, with 131 prescribing applications approved in 2022, compared to only 7 in 2019 [57].

## 6. Tourette Syndrome

Medications used to treat Tourette syndrome (TS) include alpha agonists (e.g., clonidine), and antipsychotics (e.g., risperidone). The efficacy of these medications for reduction in tics is variable, but in general, effect sizes are only modest [58]. Furthermore, these medications carry a risk of serious adverse effects including sedation or bradycardia (alpha agonists), and weight gain, extrapyramidal side effects, tardive dyskinesia, and QTC prolongation (anti-psychotics). MC is one of a number of new agents being investigated as potential treatments for TS.

The endocannabinoid system has also been implicated in the pathogenesis of Tourette’s syndrome [9,10], and thus MC has been identified as a potential therapy. Unlike other developmental and mental health disorders for which CBD-predominant products are being investigated, Δ^9^-tetrahydrocannabinol (THC) has been proposed as the key therapeutic agent in treating Tourette’s syndrome. Preliminary evidence from case studies in adolescents [59,60,61,62] and small randomised trials in adults [63,64,65] suggests that THC may be effective in reducing tics. However, high-quality studies in adolescents with Tourette’s syndrome are needed.

There are three registered trials of MC for children and adolescents with Tourette’s syndrome. An open-label pilot study will investigate MC (THC:CBD 10:15 oil) in 10 adolescents aged 12 to 18 years old, with the aim of assessing study design feasibility and acceptability for a full-scale RCT (ANZCTR: ACTRN12622000031763). A related randomised, double-blind, placebo-controlled cross-over pilot study of 10 participants will administer MC (THC:CBD 10:15 oil, THC dose up to 20 mg/day) and placebo over two 10-week treatment periods to 10 adolescents aged 12–18 years with Tourette’s syndrome. The primary objective of this pilot study is to evaluate all elements of the study design (recruitment strategy, study duration, study procedures, study medication tolerance and outcome measures) to assess if they are acceptable and feasible for the conduct of a full-scale randomised controlled trial of THC:CBD 10:15 oil to reduce tic severity in adolescents with TS (Clinicaltrials.gov: NCT05184478). Finally, a randomised, double-blind, placebo-controlled cross-over trial will assess the efficacy of CBD (Epidiolex) as a treatment of anxiety in 40 children and adolescents aged 6–17 years with Tourette’s syndrome. Secondary outcomes include measures of tic symptoms, depression, obsessive compulsive symptoms, problem behaviours and improved quality of life, sleep and global symptomology (ANZCTR: ACTRN12621001659897).

## 7. Mental Health Disorders

The potential for MC to treat a range of adult mental health disorders is beginning to be understood [66], and there is increasing interest in MC as a treatment for paediatric mental health disorders.

In a small double-blind placebo controlled trial (*n* = 37), adolescents aged 18 to 19 with social anxiety disorder and avoidant personality disorder who received CBD (300 mg per day) reported significantly lower social anxiety symptoms relative to those who received placebo after 4 weeks of treatment [67]. 

Results have recently been published from a pilot open-label study of CBD in 31 young people aged 12–25 years old with treatment-resistant anxiety [68]. Dosing commenced at 200 mg/day and followed a fixed-flexible schedule; doses were increased by 200 mg/day if participants did not show clinically meaningful improvement, up to a maximum of 800 mg/day. A total of 19 participants up-titrated to the maximum dose, while 9 reached 600 mg/day and 1 received 400 mg/day. Two participants withdrew. After 12 weeks of treatment, there was a significant decrease in anxiety, with a mean change of −42.6% in the primary outcome measure. Improvements were also noted in secondary outcome measures of anxiety, depression, and social and occupational functioning. On the Clinicians’ Global Impressions scale, 87% improved, and 53% improved substantially. There were no serious adverse events, and reported mild or moderate adverse events included fatigue, low mood, appetite change, drowsiness, nausea, diarrhea, dry-mouth, insomnia, and hot flushes or cold chills. One withdrawal was due to a skin rash. The study team have received funding for a large-scale definitive trial within this population. 

There is also interest in MC for the treatment of psychosis, with two small trials of CBD in adults suggesting CBD is well tolerated in this patient group, may be associated with a reduction in psychotic symptoms [69], and may partially normalise physiological functioning in brain regions associated with psychosis [70]. There are currently no published trials investigating the role of CBD in treating psychotic symptoms in young people. A planned three-arm randomised placebo-controlled trial of CBD (600 mg or 1000 mg per day or placebo) to treat youth (aged 12–25) at ultra-high risk of psychosis will aim to reduce positive psychotic symptoms (ANZCTR: ACTRN12621000349842) [71].

Lastly, an open label trial is underway of CBD oral capsules (up to 800 mg/day) in adolescents aged 12–18 years with anorexia nervosa, with a primary outcome of weight gain, and secondary outcomes of eating disorder symptoms, other mental health symptoms and quality of life (ANZCTR: ACTRN12622001306707).

## 8. General Considerations

### 8.1. Safety

CBD appears to have a relatively mild safety profile [72]. The most frequently reported adverse effects from trials in paediatric epilepsy have been fatigue, sedation, nausea, diarrhoea and appetite suppression [73]. It is notable that these studies mostly used very high doses, up to 20 mg/kg/day. 

In contrast, it is known that habitual recreational use of THC can cause paranoia, hallucinations and psychosis, and chronic long-term use in adolescents may affect neurodevelopmental functions such as memory and cognition, although the evidence remains uncertain [74]. Very little is known, however, about the side-effect profile of prescribed THC, particularly for children and adolescents. Although prescribed MC is likely to involve lower doses of THC than exposure from illicit cannabis use, the potential adverse effects of THC need to be considered if it is to be prescribed for young people, particularly in patients with a personal or close family history of psychotic symptoms.

### 8.2. Patient Access to Medicinal Cannabis

Patient access to prescribed MC varies in different countries. In Australia, doctors have been able to prescribe MC for the past 7 years. This requires applying for individual patient approval from the regulator, the Therapeutic Goods Administration. In the submission, the prescribing doctor must specify the clinical indication, provide a clinical justification, describe the monitoring plan, and select from one of five categories with different proportions of total non-CBD cannabinoids, as well as specifying the type of formulation to be prescribed. Some GPs, paediatricians and child and adolescent psychiatrists have begun prescribing MC products for children and adolescents with developmental and mental health disorders. In 2022, 1783 applications to prescribe MC for patients under age 18 years were approved, the vast majority being for liquid preparations (oils) [57]. The most common clinical indications were ASD (575), anxiety (503), epilepsy (215) and ADHD (131). 

All MC preparations are very expensive, with cost representing a likely barrier to access for many patients. Furthermore, anecdotally, many doctors are unwilling to prescribe MC, reporting that they lack knowledge about the evidence for benefits and safety, and also that they are unfamiliar with the prescribing process, which is more complicated than prescribing other medications. 

Use of non-prescribed (unregulated) MC preceded use of prescribed MC, and non-prescribed MC continues to be used for a range of reasons, including ease of access and cost. However, both overlabelling and underlabelling of the content of the major cannabinoids THC and CBD has been identified in investigations of labelling accuracy of MC products, introducing the risks of lack of benefit or adverse events [75,76]. Furthermore, there is a risk of drug interactions with the use of non-prescribed MC. 

### 8.3. Attitudes of Parents and Health Professionals toward MC for Children

In a recent large US nationally representative household survey, 73% of parents of children aged 3–18 years indicated they believed CBD may be a good option for children when other medications do not work [77]. Among a US-based sample of children with ASD receiving MC, four out of five carers indicated that it was chosen as a therapy because it is a natural remedy [78]. We have recently conducted a large survey of the attitudes of Australian parents of children with developmental, behavioural and/or emotional problems to MC (manuscript in preparation). Over three quarters reported that they would be comfortable giving it to their child. A paired survey of paediatricians and child psychiatrists found that three quarters had been asked about MC by a parent, and over half believed it was a legitimate medical therapy. Anecdotally, some parents have requested to try MC (particularly CBD) for their child first line, i.e., before other psychotropic medications, because they believe it is less likely to cause adverse effects.

### 8.4. Research Challenges

The establishment and conduct of clinical trials of MC often present greater challenges compared to trials of other drugs. These include limited preclinical and safety data, limited information to inform choice of MC product and dose, sourcing a continuous supply of high-quality investigational product with reliable batch-to-batch consistency, manufacture of a quality placebo, limitations on shelf-life, and requirements for secure storage [79]. Furthermore, the high cost of MC generates unusually large study budgets. These factors can result in difficulties in obtaining grant funding, ethics approvals and governance certificates, and in successfully carrying trials through to completion. 

## 9. Conclusions

Evidence to inform the appropriate use of MC in the treatment of paediatric developmental, behavioural and mental health disorders is gradually emerging. A variety of products and doses have been used in different trials. Results of studies in ASD, the disorder most studied to date, have been mixed. Synthetic, transdermal CBD gel has demonstrated efficacy for reducing social avoidance in a sub-group of children with Fragile X syndrome. Trials are planned or underway in ASD, intellectual disability, several specific neurodevelopmental syndromes, Tourette’s syndrome, anxiety, anorexia nervosa and psychosis. There are currently no trials completed or underway to support prescribing in ADHD. Should current trials support the use of MC in children, then in order to guide clinical prescribing, future research may seek to investigate the optimal therapeutic dose range and cannabinoid profile—for example, the most effective ratio of CBD and THC—or the potential role of minor cannabinoids.

Despite the lack of good evidence, prescribing of MC for these patients is increasing. Professional organisations have urged caution in prescribing MC until there is stronger supportive evidence [80,81].

## Figures and Tables

**Table 1:** Published studies of medicinal cannabis in autism spectrum disorder.

Reference	Study Design	Population	Product Details	Findings
Aran et al., 2019 [26]	Retrospective	60 children aged 5–17 years with ASD and severe behaviour disturbance	Initial product contained whole plant extract CBD and THC in a 20:1 ratio. 29 patients with an insufficient response commenced strains with a CBD/THC ratio up to 6:1. Mean total daily dose was 3.8 mg/kg/day CBD and 0.29 mg/kg/day THC for those taking three daily doses (*n* = 44), and 1.8 mg/kg/day CBD and 0.22 mg/kg/day THC for those taking two daily doses (*n* = 16)	“Much improved” or “very much improved”: behaviour 61%, anxiety 39%, communication in 47%. (p. 1286) Adverse events included sleep disturbances (14%) irritability (9%) and loss of appetite (9%). (p. 1285) One serious adverse event was noted, a transient psychotic event, which was considered related to the THC content.
Aran et al., 2021 [33]	Placebo-controlled double-blind comparison of two oral cannabinoid solutions	150 participants with ASD, aged 5–21 years	(1) Whole-plant cannabis extract containing CBD and THC at a 20:1 ratio and (2) Purified CBD and THC at 20:1 ratio. Average treatment dose was 5.7 mg/kg/d of CBD in the whole-plant extract arm and 5.9 mg/kg/d of CBD in the pure cannabinoid arm.	Disruptive behaviour (co-primary outcome, measured on the Clinical Global Impression-Improvement scale) was much or very much improved in 49% of participants in the whole plant extract group compared to 21% in the placebo group (*p* = 0.005). In the pure cannabinoid group, 38% were rated as much or very much improved, which was not significant compared to placebo (*p* = 0.08). (p. 6) No significant difference was found between the groups in changes on the other co-primary-outcome measure of noncompliant behaviour or the secondary-outcome measure of parenting stress. Median social responsiveness score (secondary outcome) improved by 14.9 points on whole-plant extract versus 3.6 after placebo (*p* = 0.009). Common adverse events included somnolence, decreased appetite, weight loss, tiredness, euphoria and anxiety. No serious adverse events were reported. (p. 7)
Barchel et al., 2019 [27]	Prospective	53 youths with ASD, aged 4–22 years	CBD:THC in 20:1 ratio. Individualised dose: median (IQR) CBD daily dose was 90 (45–143) mg	Overall improvement was reported in 75% of participants. (p. 3) Changes in the following symptoms were reported (improved, worsened): self-injury and rage attacks 68%; 9%; hyperactivity 68%, 3%; sleep 71, 5%; anxiety 47%, 24%. Adverse effects were described as mild; most common were somnolence and decreased appetite.
Bar-Lev Schleider et al., 2018 [31]	Prospective open-label	During the study period, 188 patients with ASD initiated the treatment. Mean age was 12.9 ± 7.0 years.	Products varied—most patients received 30% CBD/1.5% THC. Mean daily dose was CBD 240 mg and THC 12 mg.	After one month, 179 patients remained on treatment and data were collected from 119:49% reported significant improvement, 31% reported moderate improvement, and 14% reported no improvement. (pp. 2–3) The most common symptoms improved were restlessness, rage attacks and agitation. The most common adverse effects were restlessness and sleepiness.
Bilge & Ekici 2021 [28]	Retrospective	33 patients with ASD, mean age 7.7 ± 5.5 years	Two CBD-enriched cannabis brands were used; both similar full spectrum CBD with trace THC. Average daily CBD-enriched cannabis dose was 0.7 mg/kg (0.3–2 mg/kg). Maximum daily maintenance dose 40 mg/day.	According to the parents’ reports: no change in daily life activity was reported in 6 (19.35%) patients. (p. 4) Improvements included: decrease in behavioral problems (32.2%), increase in expressive language (22.5%), improved cognition (12.9%), increase in social interaction (9.6%). Adverse events included restlessness (*n* = 7), generalised seizures (*n* = 1) and increased stereotypies (*n* = 1).
Fleury-Teixeira et al., 2019 [29]	Observational	18 patients with ASD aged 6–17 years; data collected from 15 who adhered to the treatment	Oral ~75/1 CBD/THC. Individualised titration: Average initial dose of CBD was ~2.90 mg/kg/day, average dose at end of the study was 4.55 mg/kg/day (range: 3.75 to 6.45 mg/kg/day)	3 patients withdrew within one month due to adverse effects. (p. 4) Of the 15 patients who adhered to treatment, only one patient showed no improvement in symptoms of ASD. (p. 5) Improvements were most pronounced in sleep disorders, seizures, and behavioral crisis. Signs of improvement were reported for motor development, communication and social interaction, and cognitive performance Among the 15 patients who adhered to treatment, the following mild and/or transient adverse effects were reported: sleepiness, moderate irritability (three cases each); diarrhea, increased appetite, conjunctival hyperemia, and increased body temperature (one case each).
Hacohen et al., 2022 [30]	Prospective open-label	110 participants with ASD recruited. Data analysed from 82 who completed the 6-month study period. Mean age: 9.2 years (range: 5–25 years).	Whole-plant extract in oil with a CBD:THC ratio of 20:1, starting at one drop daily (each drop contains: 0.3 mg THC and 5.7 mg CBD) and gradually increasing until parents perceived improvements in their child’s behaviour. Maximum dosage was 10 mg/kg/day (or total of 400 mg/day) of CBD and 0.5 mg/kg/day (or total of 20 mg/day) of THC.	28 of 110 subjects were withdrawn from treatment due to inability to follow the protocol (*n* = 8), adverse events (*n* = 12, including increased aggression, anxiety or hyperactivity, weight gain, abdominal pain, and decreased communication), and lack of improvement (*n* = 8). (p. 3) In those who completed end-of-treatment assessments, significant improvement was reported in ASD symptoms (social affect on the ADOS, and social behaviour and restricted repetitive behaviour on the SRS), as well as adaptive function (communication, daily living skills and socialisation). (p. 4) Median change in ADOS social affect scores was zero, suggesting that at least half of participants who completed treatment did not report improvement on this measure. (p. 5) Significant change (either improvement or deterioration) was not reported on cognitive function tasks.

## Data Availability

Not applicable.

## Supplementary Materials

The following supporting information can be downloaded at: https://www.mdpi.com/article/10.3390/ijerph20085430/s1, Table S1: Registered trials of medicinal cannabis in autism spectrum disorder.

## References

[1] Efron D., Freeman J. (2018). Medical cannabis for paediatric developmental–behavioural and psychiatric disorders. J. Paediatr. Child Health.

[2] Sheehan R., Strydom A., Morant N., Pappa E., Hassiotis A. (2017). Psychotropic prescribing in people with intellectual disability and challenging behaviour. BMJ.

[3] Turner M. (2020). The role of drugs in the treatment of autism. Aust. Prescr..

[4] Efron D., Danchin M.H., Cranswick N.E., Gulenc A., Hearps S., Hiscock H. (2017). Medication prescribed by Australian paediatricians: Psychotropics predominate. J. Paediatr. Child Health.

[5] Garland E.J., Kutcher S., Virani A., Elbe D. (2016). Update on the use of SSRIs and SNRIs with children and adolescents in clinical practice. J. Can. Acad. Child Adolesc. Psychiatry.

[6] Vitiello B., Correll C., van Zwieten-Boot B., Zuddas A., Parellada M., Arango C. (2009). Antipsychotics in children and adolescents: Increasing use, evidence for efficacy and safety concerns. Eur. Neuropsychopharmacol..

[7] Chakrabarti B., Persico A., Battista N., Maccarrone M. (2015). Endocannabinoid signaling in autism. Neurotherapeutics.

[8] Földy C., Malenka R.C., Südhof T.C. (2013). Autism-associated neuroligin-3 mutations commonly disrupt tonic endocannabinoid signaling. Neuron.

[9] Müller-Vahl K.R., Bindila L., Lutz B., Musshoff F., Skripuletz T., Baumgaertel C., Sühs K.-W. (2020). Cerebrospinal fluid endocannabinoid levels in Gilles de la Tourette syndrome. Neuropsychopharmacology.

[10] Szejko N., Fichna J.P., Safranow K., Dziuba T., Żekanowski C., Janik P. (2020). Association of a variant of CNR1 gene encoding cannabinoid receptor 1 with gilles de la Tourette syndrome. Front. Genet..

[11] Palumbo J.M., Thomas B.F., Budimirovic D., Siegel S., Tassone F., Hagerman R., Faulk C., O’Quinn S., Sebree T. (2023). Role of the endocannabinoid system in fragile X syndrome: Potential mechanisms for benefit from cannabidiol treatment. J. Neurodev. Disord..

[12] Hughes H.K., Mills Ko E., Rose D., Ashwood P. (2018). Immune dysfunction and autoimmunity as pathological mechanisms in autism spectrum disorders. Front. Cell. Neurosci..

[13] Poleg S., Golubchik P., Offen D., Weizman A. (2018). Cannabidiol as a suggested candidate for treatment of autism spectrum disorder. Prog. Neuropsychopharmacol. Biol. Psychiatry.

[14] Blessing E.M., Steenkamp M.M., Manzanares J., Marmar C.R. (2015). Cannabidiol as a potential treatment for anxiety disorders. Neurotherapeutics.

[15] Marsicano G., Lutz B. (1999). Expression of the cannabinoid receptor CB1 in distinct neuronal subpopulations in the adult mouse forebrain. Eur. J. Neurosci..

[16] Kibret B.G., Canseco-Alba A., Onaivi E.S., Engidawork E. (2023). Crosstalk between the endocannabinoid and mid-brain dopaminergic systems: Implication in dopamine dysregulation. Front. Behav. Neurosci..

[17] Devinsky O., Cross J.H., Laux L., Marsh E., Miller I., Nabbout R., Scheffer I.E., Thiele E.A., Wright S. (2017). Trial of cannabidiol for drug-resistant seizures in the Dravet syndrome. N. Engl. J. Med..

[18] Devinsky O., Marsh E., Friedman D., Thiele E., Laux L., Sullivan J., Miller I., Flamini R., Wilfong A., Filloux F. (2016). Cannabidiol in patients with treatment-resistant epilepsy: An open-label interventional trial. Lancet Neurol..

[19] Devinsky O., Patel A.D., Cross J.H., Villanueva V., Wirrell E.C., Privitera M., Greenwood S.M., Roberts C., Checketts D., VanLandingham K.E. (2018). Effect of cannabidiol on drop seizures in the Lennox–Gastaut syndrome. N. Engl. J. Med..

[20] Press C.A., Knupp K.G., Chapman K.E. (2015). Parental reporting of response to oral cannabis extracts for treatment of refractory epilepsy. Epilepsy Behav..

[21] Zeidan J., Fombonne E., Scorah J., Ibrahim A., Durkin M.S., Saxena S., Yusuf A., Shih A., Elsabbagh M. (2022). Global prevalence of autism: A systematic review update. Autism Res..

[22] Joshi G., Petty C., Wozniak J., Henin A., Fried R., Galdo M., Kotarski M., Walls S., Biederman J. (2010). The heavy burden of psychiatric comorbidity in youth with autism spectrum disorders: A large comparative study of a psychiatrically referred population. J. Autism Dev. Disord..

[23] Newcomb E.T., Hagopian L.P. (2018). Treatment of severe problem behaviour in children with autism spectrum disorder and intellectual disabilities. Int. Rev. Psychiatry.

[24] Soke G.N., Rosenberg S.A., Rosenberg C.R., Vasa R.A., Lee L.-C., DiGuiseppi C. (2018). Self-injurious behaviors in children with autism spectrum disorder enrolled in the study to explore early development. Autism.

[25] Madden J.M., Lakoma M.D., Lynch F.L., Rusinak D., Owen-Smith A.A., Coleman K.J., Quinn V.P., Yau V.M., Qian Y.X., Croen L.A. (2017). Psychotropic medication use among insured children with autism spectrum disorder. J. Autism Dev. Disord..

[26] Aran A., Cassuto H., Lubotzky A., Wattad N., Hazan E. (2019). Brief Report: Cannabidiol-Rich Cannabis in Children with Autism Spectrum Disorder and Severe Behavioral Problems—A Retrospective Feasibility Study. J. Autism Dev. Disord..

[27] Barchel D., Stolar O., De-Haan T., Ziv-Baran T., Saban N., Fuchs D.O., Koren G., Berkovitch M. (2019). Oral cannabidiol use in children with autism spectrum disorder to treat related symptoms and co-morbidities. Front. Pharmacol..

[28] Bilge S., Ekici B. (2021). CBD-enriched cannabis for autism spectrum disorder: An experience of a single center in Turkey and reviews of the literature. J. Cannabis Res..

[29] Fleury-Teixeira P., Caixeta F.V., Ramires da Silva L.C., Brasil-Neto J.P., Malcher-Lopes R. (2019). Effects of CBD-enriched cannabis sativa extract on autism spectrum disorder symptoms: An observational study of 18 participants undergoing compassionate use. Front. Neurol..

[30] Hacohen M., Stolar O.E., Berkovitch M., Elkana O., Kohn E., Hazan A., Heyman E., Sobol Y., Waissengreen D., Gal E. (2022). Children and adolescents with ASD treated with CBD-rich cannabis exhibit significant improvements particularly in social symptoms: An open label study. Transl. Psychiatry.

[31] Bar-Lev Schleider L., Mechoulam R., Saban N., Meiri G., Novack V. (2019). Real life Experience of Medical Cannabis Treatment in Autism: Analysis of Safety and Efficacy. Sci. Rep..

[32] Silva Junior E.A.d., Medeiros W.M.B., Torro N., Sousa J.M.M.d., Almeida I.B.C.M.d., Costa F.B.d., Pontes K.M., Nunes E.L.G., Rosa M.D.d., Albuquerque K.L.G.D.d. (2021). Cannabis and cannabinoid use in autism spectrum disorder: A systematic review. Trends Psychiatry Psychother..

[33] Aran A., Harel M., Cassuto H., Polyansky L., Schnapp A., Wattad N., Shmueli D., Golan D., Castellanos F.X. (2021). Cannabinoid treatment for autism: A proof-of-concept randomized trial. Mol. Autism.

[34] Schnapp A., Harel M., Cayam-Rand D., Cassuto H., Polyansky L., Aran A. (2022). A Placebo-Controlled Trial of Cannabinoid Treatment for Disruptive Behavior in Children and Adolescents with Autism Spectrum Disorder: Effects on Sleep Parameters as Measured by the CSHQ. Biomedicines.

[35] Whitehall J. (2022). Medical marijuana: A Triumph of hope over experience. Quadrant.

[36] Thomas M., Frampton C. HOPE® 1 Demonstrates Improvements in Clinical Global Impression (CGI) in Patients with Autism Spectrum Disorder. https://zeliratx.com/wp-content/uploads/2022/04/ZEL040-White-Paper_Hope_FA.pdf.

[37] Fairhurst C., Kumar R., Checketts D., Tayo B., Turner S. (2020). Efficacy and safety of nabiximols cannabinoid medicine for paediatric spasticity in cerebral palsy or traumatic brain injury: A randomized controlled trial. Dev. Med. Child Neurol..

[38] Palumbo J.M., Heussler H., Duhig M.J., Hurst T., O’Neill C., Sebree T. (2022). Longer-term Tolerability and Efficacy of ZYN002 Cannabidiol Transdermal Gel in Children and Adolescents with Autism Spectrum Disorder: An Open-label Phase 2 Study (BRIGHT [ZYN2-CL-030]). Pediatrics.

[39] Zamberletti E., Rubino T., Parolaro D. (2021). Therapeutic potential of cannabidivarin for epilepsy and autism spectrum disorder. Pharmacol. Ther..

[40] Tonge B.J., Einfeld S.L. (2003). Psychopathology and intellectual disability: The Australian child to adult longitudinal study. Int. Rev. Res. Ment. Retard..

[41] Sheehan R., Horsfall L., Strydom A., Osborn D., Walters K., Hassiotis A. (2017). Movement side effects of antipsychotic drugs in adults with and without intellectual disability: UK population-based cohort study. BMJ Open.

[42] Kruger T., Christophersen E. (2006). An open label study of the use of dronabinol (Marinol) in the management of treatment-resistant self-injurious behavior in 10 retarded adolescent patients. J. Dev. Behav. Pediatr..

[43] Efron D., Taylor K., Freeman J., Cranswick N.E., Payne J.M., Mulraney M., Prakash C., Lee K., Williams K. (2020). Does cannabidiol reduce severe behavioural problems in children with intellectual disability? Study protocol for a pilot single-site phase I/II randomised placebo controlled trial. BMJ Open.

[44] Heussler H., Cohen J., Silove N., Tich N., Bonn-Miller M.O., Du W., O’Neill C., Sebree T. (2019). A phase 1/2, open-label assessment of the safety, tolerability, and efficacy of transdermal cannabidiol (ZYN002) for the treatment of pediatric fragile X syndrome. J. Neurodev. Disord..

[45] Berry-Kravis E., Hagerman R., Budimirovic D., Erickson C., Heussler H., Tartaglia N., Cohen J., Tassone F., Dobbins T., Merikle E. (2022). A randomized, controlled trial of ZYN002 cannabidiol transdermal gel in children and adolescents with fragile X syndrome (CONNECT-FX). J. Neurodev. Disord..

[46] Polanczyk G.V., Willcutt E.G., Salum G.A., Kieling C., Rohde L.A. (2014). ADHD prevalence estimates across three decades: An updated systematic review and meta-regression analysis. Int. J. Epidemiol..

[47] Efron D., Davies S., Sciberras E. (2013). Current Australian pediatric practice in the assessment and treatment of ADHD. Acad. Pediatr..

[48] Visser S.N., Danielson M.L., Bitsko R.H., Holbrook J.R., Kogan M.D., Ghandour R.M., Perou R., Blumberg S.J. (2014). Trends in the parent-report of health care provider-diagnosed and medicated attention-deficit/hyperactivity disorder: United States, 2003–2011. J. Am. Acad. Child Adolesc. Psychiatry.

[49] Rappley M.D. (2005). Attention Deficit–Hyperactivity Disorder. N. Engl. J. Med..

[50] Sinha D., Efron D. (2005). Complementary and alternative medicine use in children with attention deficit hyperactivity disorder. J. Paediatr. Child Health.

[51] Francisco A.P., Lethbridge G., Patterson B., Bergmann C.G., Van Ameringen M. (2022). Cannabis use in attention–Deficit/hyperactivity disorder (ADHD): A scoping review. J. Psychiatr. Res..

[52] Lee S.S., Humphreys K.L., Flory K., Liu R., Glass K. (2011). Prospective association of childhood attention-deficit/hyperactivity disorder (ADHD) and substance use and abuse/dependence: A meta-analytic review. Clin. Psychol. Rev..

[53] Mitchell J.T., Sweitzer M.M., Tunno A.M., Kollins S.H., McClernon F.J. (2016). “I use weed for my ADHD”: A qualitative analysis of online forum discussions on cannabis use and ADHD. PLoS ONE.

[54] Stueber A., Cuttler C. (2022). Self-reported effects of cannabis on ADHD symptoms, ADHD medication side effects, and ADHD-related executive dysfunction. J. Atten. Disord..

[55] Cooper R.E., Williams E., Seegobin S., Tye C., Kuntsi J., Asherson P. (2017). Cannabinoids in attention-deficit/hyperactivity disorder: A randomised-controlled trial. Eur. Neuropsychopharmacol..

[56] Mansell H., Quinn D., Kelly L.E., Szafron M., Alcorn J. (2021). Pharmacokinetics and Perceptions of Children and Young Adults Using Cannabis for Attention-Deficit/Hyperactivity Disorder and Oppositional Defiant Disorder: Protocol for a Mixed Methods Proof-of-Concept Study. JMIR Res. Protoc..

[57] Therapeutic Goods Administration Department of Health and Aged Care Australian Government Medicinal Cannabis Special Access Scheme Category B Data. https://www.tga.gov.au/products/unapproved-therapeutic-goods/medicinal-cannabis-hub/medicinal-cannabis-access-pathways-and-patient-access-data/medicinal-cannabis-special-access-scheme-category-b-data.

[58] Besag F.M., Vasey M.J., Lao K.S., Chowdhury U., Stern J.S. (2021). Pharmacological treatment for Tourette syndrome in children and adults: What is the quality of the evidence? A systematic review. J. Psychopharmacol..

[59] Hasan A., Rothenberger A., Münchau A., Wobrock T., Falkai P., Roessner V. (2010). Oral Δ9-tetrahydrocannabinol improved refractory Gilles de la Tourette syndrome in an adolescent by increasing intracortical inhibition: A case report. J. Clin. Psychopharmacol..

[60] Jakubovski E., Müller-Vahl K. (2017). Speechlessness in Gilles de la Tourette syndrome: Cannabis-based medicines improve severe vocal blocking tics in two patients. Int. J. Mol. Sci..

[61] Szejko N., Jakubovski E., Fremer C., Kunert K., Müller-Vahl K. (2018). Delta-9-tetrahydrocannabinol for the treatment of a child with Tourette syndrome: Case report. Eur. J. Med. Case Rep..

[62] Szejko N., Jakubovski E., Fremer C., Müller-Vahl K.R. (2019). Vaporized cannabis is effective and well-tolerated in an adolescent with Tourette syndrome. Med. Cannabis Cannabinoids.

[63] Müller-Vahl K.R., Schneider U., Koblenz A., Jöbges M., Kolbe H., Daldrup T., Emrich H. (2002). Treatment of Tourette's syndrome with Δ9-tetrahydrocannabinol (THC): A randomized crossover trial. Pharmacopsychiatry.

[64] Abi-Jaoude E., Bhikram T., Parveen F., Levenbach J., Lafreniere-Roula M., Sandor P. (2022). A Double-Blind, Randomized, Controlled Crossover Trial of Cannabis in Adults with Tourette Syndrome. Cannabis Cannabinoid Res..

[65] Müller-Vahl K.R., Schneider U., Prevedel H., Theloe K., Kolbe H., Emrich H.M., Daldrup T. (2003). Delta 9-tetrahydrocannabinol (THC) is effective in the treatment of tics in Tourette syndrome: A 6-week randomized trial. J. Clin. Psychiatry.

[66] Sarris J., Sinclair J., Karamacoska D., Davidson M., Firth J. (2020). Medicinal cannabis for psychiatric disorders: A clinically-focused systematic review. BMC Psychiatry.

[67] Masataka N. (2019). Anxiolytic effects of repeated cannabidiol treatment in teenagers with social anxiety disorders. Front. Psychol..

[68] Berger M., Li E., Rice S., Davey C.G., Ratheesh A., Adams S., Jackson H., Hetrick S., Parker A., Spelman T. (2022). Cannabidiol for treatment-resistant anxiety disorders in young people: An open-label trial. J. Clin. Psychiatry.

[69] Leweke F., Piomelli D., Pahlisch F., Muhl D., Gerth C., Hoyer C., Klosterkötter J., Hellmich M., Koethe D. (2012). Cannabidiol enhances anandamide signaling and alleviates psychotic symptoms of schizophrenia. Transl. Psychiatry.

[70] Bhattacharyya S., Wilson R., Appiah-Kusi E., O’Neill A., Brammer M., Perez J., Murray R., Allen P., Bossong M.G., McGuire P. (2018). Effect of cannabidiol on medial temporal, midbrain, and striatal dysfunction in people at clinical high risk of psychosis: A randomized clinical trial. JAMA Psychiatry.

[71] Amminger G.P., Lin A., Kerr M., Weller A., Spark J., Pugh C., O’Callaghan S., Berger M., Clark S.R., Scott J.G. (2022). Cannabidiol for at risk for psychosis youth: A randomized controlled trial. Early Interv. Psychiatry.

[72] Iffland K., Grotenhermen F. (2017). An update on safety and side effects of cannabidiol: A review of clinical data and relevant animal studies. Cannabis Cannabinoid Res..

[73] Raucci U., Pietrafusa N., Paolino M.C., Di Nardo G., Villa M.P., Pavone P., Terrin G., Specchio N., Striano P., Parisi P. (2020). Cannabidiol treatment for refractory epilepsies in pediatrics. Front. Pharmacol..

[74] Jackson N.J., Isen J.D., Khoddam R., Irons D., Tuvblad C., Iacono W.G., McGue M., Raine A., Baker L.A. (2016). Impact of adolescent marijuana use on intelligence: Results from two longitudinal twin studies. Proc. Natl. Acad. Sci. USA.

[75] Suraev A., Lintzeris N., Stuart J., Kevin R., Blackburn R., Richards E., Arnold J., Ireland C., Todd L., Allsop D. (2018). Composition and use of cannabis extracts for childhood epilepsy in the Australian community. Sci. Rep..

[76] Vandrey R., Raber J.C., Raber M.E., Douglass B., Miller C., Bonn-Miller M.O. (2015). Cannabinoid dose and label accuracy in edible medical cannabis products. JAMA.

[77] C.S. Mott Children’s Hospital National Poll on Children’s Health: Parent Perspectives on CBD Use in Children. https://mottpoll.org/sites/default/files/documents/022122_CBD.pdf.

[78] DiLiberto M.A., Zuppa A.F., Cornetta A., Faig W., Scully T., Bennett A., Thomas M., Ward E., Barr S., Yerys B.E. (2022). A natural history study of medical cannabis consumption in pediatric autism in the United States. Res. Autism Spectr. Disord..

[79] Martin J.H., Hill C., Walsh A., Efron D., Taylor K., Kennedy M., Galettis R., Lightfoot P., Hanson J., Irving H. (2020). Clinical trials with cannabis medicines—Guidance for ethics committees, governance officers and researchers to streamline ethics applications and ensuring patient safety: Considerations from the Australian experience. Trials.

[80] Wong S.S., Wilens T.E. (2017). Medical cannabinoids in children and adolescents: A systematic review. Pediatrics.

[81] Martin J.H., Bonomo Y., Reynolds A.D. (2018). Compassion and evidence in prescribing cannabinoids: A perspective from the Royal Australasian College of Physicians. Med. J. Aust..

