# Supplementary material for: Medicinal Cannabis for Paediatric Developmental, Behavioural and Mental Health Disorders
Source: Int J Environ Res Public Health. 2023 Apr 7;20(8):5430. doi: 10.3390/ijerph20085430 (PMC10138057; doi:10.3390/ijerph20085430)
Supplement: Supplementary file 1 [file ijerph-20-05430-s001.zip › ijerph-2245079-supplementary.pdf]

Supplementary Table S1: Registered trials of medicinal cannabis in autism spectrum disorder

Registry Number	Location	Design	Population	Product	Outcomes
ACTRN12622000437763	Australia	Double-blind, randomised crossover trial of CBD vs placebo	34 participants with ASD, Age 5-12 years, IQ >40	10 mg/kg/day of cannabidiol (Medigrowth CBD100 100mg/ml CBD, <1mg/ml THC) for 12 weeks	Primary outcome: Social Responsiveness Scale<br><br>Secondary outcome: EEG during social task; behavioural and QOL questionnaires.
ACTRN12621000760875	Australia	Prospective open label	20 participants with ASD aged 8 to 17	"NTI164" Full-spectrum medicinal cannabis plant extract with 0.08% THC - Children will receive the maximum tolerated dose daily or 20mg/kg/day	Primary outcome: Clinical Global Impression Scale – Improvement (CGI-I); adaptive behaviour functioning; safety (assessed by full blood examination).<br><br>Secondary outcome: behavioural, mental health and QOL questionnaires; safety as assessed by liver and kidney function blood tests.
ACTRN12621000370808	Australia	Open label, observational study	150 participants with ASD aged 6 and older (includes adults)	Zelira Hope products: Hope 1 (CBD:THC 1:1) and Hope 2 (CBD:THC	Behaviour difficulties, clinician and caregiver

				1:5), dose as clinically indicated based on effectiveness and tolerability	rating of change, sleep, parenting stress.
NCT05626959	Australia	Double-Blind, Randomised and Controlled-to-Open-Label Study	54 participants with ASD, aged 8 to 17 years	"NTI164" Full-Spectrum Medicinal Cannabis Plant Extract with less than 0.08% THC, with dosage up to 20mg/kg/day	Primary outcome: change as rated on the CGI.<br>Secondary outcomes: adaptive function, social responsiveness, mental health symptoms, sleep.
NCT04517799	USA	Double-Blind, Crossover Trial	42 males with ASD aged 7-14 years old	Epidiolex (CBD isolate) 20mg/kg/day – 8 week intervention periods	Primary outcome: repetitive behaviour, problem behaviour and ASD symptoms.<br>Secondary: aberrant behaviour, self-harm, social responsiveness, vocabulary, sleep
NCT04520685	USA	Randomized, placebo-controlled study.<br>Group A will receive cannabidiol for the first 12 weeks of the study	70 participants with ASD aged 5-17 years old	Cannabidiol 100mg/ml, target dose 10mg/kg/day.	Primary outcome: irritability.<br>Secondary: behaviour problems, anxiety, depression, mood,

		and placebo for the last 15 weeks.<br><br>Group B will receive placebo for the first 15 weeks of the study and cannabidiol for the last 12 weeks<br><br>Group C will receive cannabidiol throughout the entire 27 weeks of treatment			social responsiveness, behaviour, sleep, QOL
NCT04745026	International – UK, USA, Australia	Randomized, Double-blind, Placebo-controlled Trial	160 participants with ASD and IQ > 70, aged 6-17 years	GW Epidiolex 10mg/kg/day	Primary: Behaviour problems, adaptive function, CGI
NCT03900923	USA	Open, Bayesian optimal interval (BOIN) design: participants were assigned to cohorts of size 3 receiving doses of 3, 6, or 9 mg/kg/day, depending on the treatment response of participants in prior cohorts	30 participants with ASD between the ages of 7 and 17 years old, IQ >80	GW Epidiolex	Primary: CGI<br><br>Secondary: range of outcomes including repetitive behaviour, social responsiveness, behaviour, anxiety, depression, mood, sleep, anxiety, adhd symptoms
NCT05212493	Israel	Open label, randomized, two active comparison arms	128 participants aged 5 to 25, diagnosed with ASD by DSM	Two cannabidiol-rich (CBD:THC ratio of 20:1) cannabis oil products	Primary: Attention, cognition, communication skills, adaptive behaviour,

			IQ below 70. The investigator can include patients with IQ above 70 if they have significant ASD or comorbid symptoms	from different manufacturers	violent behaviour, cannabinoid metabolites in participant blood.<br>Secondary: side effects, sleep, eating, hormonal profile and biochemical parameters.
NCT03202303	USA	Phase 2, 12-week double-blind, randomized, placebo-controlled trial	100 participants with ASD aged 5 to 18 years old	10 mg/kg/day of CBDV	Primary: irritable behaviour; Secondary: Behaviour, QOL, CGI
